# Chloroform in Infantile Convulsions

**Published:** 1859-01

**Authors:** 


### CHLOROFORM IN INFANTILE CONVULSIONS.

Under the head of "Selections," will be found an article of interest, upon the Pathology and treatment of Infantile Convulsions, one of the most interesting maladies in the whole range of disease.

Without entering at this time, into the field of discussion, as to the causes which induce convulsive action in children, we can most confidently recommend Cholroform by inhalation, as by far the most efficient agent in its control, which we have ever used.

The writer of the article alluded to, seems to us to have a very inadequate idea of what we believe to be the pre-eminent value of this agent in this most distressing pathological condition.

While we would not ignore other treatment which might rationally be indicated in the variety, which presents itself under the head of "Infantile Convulsions," we believe there are but very few cases in which it might not be used with propriety and success in the control of the paroxysm.

Bitterly opposed as we are to the *present indiscriminate and loose manner of using this dangerous agent*, so remarkable are the effects which we have witnessed in the relief of the deeply interesting class of sufferers, involved in this question, that we earnestly desire every practitioner to be impressed with the value of the agent alluded to, at the same time that we would urge upon all, the importance of greater care, and *more system*, in its administration.

